# Institutional Variation in MRI Utilization After Prostatectomy for Prostate Cancer: A Multicenter Study

**DOI:** 10.3390/healthcare14193131

**Published:** 2026-09-22

**Authors:** Seungjae Lee, Moon Hyung Choi, Hokun Kim, Dong Jin Chung, Su Lim Lee, Yeon Soo Lim, Hyun A Kim, Geon Park, Woojoo Lee

**Affiliations:** 1 Department of Applied Statistics, Kyonggi University, Suwon-si 16227, Republic of Korea; 2 Department of Radiology, College of Medicine, The Catholic University of Korea, Seoul 06591, Republic of Korea; 3 Institute of Health and Environment, Seoul National University, Seoul 08826, Republic of Korea; 4 Department of Public Health Sciences, Graduate School of Public Health, Seoul National University, Seoul 08826, Republic of Korea

**Keywords:** prostate neoplasm, magnetic resonance imaging, prostatectomy, follow-up, healthcare utilization, institutional variation

## Abstract

**Highlights:**

**What are the main findings?**

- Repeated postoperative MRI use varied markedly across seven institutions, even after adjustment for measured clinical and pathological factors.
- Biochemical recurrence was associated with more frequent postoperative MRI examinations, whereas grade group and pathological T stage were not significantly associated with repeated postoperative MRI use.
- MRI utilization declined substantially after postoperative year 5.

**What are the implications of the main findings?**

- The substantial institutional variation was not fully explained by the measured indicators of recurrence risk.
- Clearer evidence-based criteria and standardized surveillance pathways may promote more consistent use of postoperative MRI across institutions.

**Abstract:**

**Background/Objectives:** Healthcare utilization may vary because of institutional practice patterns and policy context. This study aimed to evaluate institutional variation and clinical factors associated with repeated MRI examinations after prostatectomy and to explore whether cumulative MRI use was associated with overall survival. **Methods:** This retrospective multicenter study included 3754 patients who underwent prostatectomy for prostate cancer at seven hospitals. Repeated MRI examinations were analyzed using an Andersen–Gill counting-process Cox model with patient-clustered robust variance, and a complementary Poisson model was used to evaluate postoperative temporal patterns and the robustness of the institutional associations. Biochemical recurrence (BCR; postoperative prostate-specific antigen ≥ 0.2 ng/mL) was treated as a time-updated covariate. A separate exploratory Cox model evaluated overall survival. **Results:** During a median follow-up of 936 days (interquartile range, 432–1687 days), 2027 postoperative MRI examinations were recorded. Repeated postoperative MRI examinations were more frequent at Hospitals 2–7 than at Hospital 1 (average hazard ratios [HRs] over follow-up, 4.083–23.136; all *p* < 0.001), although the magnitude of these institutional associations varied over postoperative time (joint hospital-by-time interaction, *p* < 0.001). MRI examinations were also more frequent after BCR (HR, 1.565; 95% confidence interval [CI], 1.371–1.786; *p* < 0.001). Grade group and pathological T stage were not significantly associated with repeated MRI use. The annual MRI rate decreased from 18.08 per 100 person-years in postoperative year 5 to 5.94 in year 6. In the exploratory survival analysis, cumulative MRI use was not significantly associated with overall survival (HR, 0.782; 95% CI, 0.564–1.086; *p* = 0.142). **Conclusions:** MRI utilization after prostatectomy varied substantially across institutions and over postoperative time, with a marked decline after postoperative year 5. Although MRI examinations were more frequent after BCR, the large institutional differences were not fully explained by the measured clinical factors. These findings highlight the need for more standardized and evidence-based criteria for MRI use after prostatectomy.

## 1. Introduction

Prostate cancer (PCa) is one of the most commonly diagnosed cancers in men, and prostatectomy is a standard curative treatment for localized disease [1,2,3,4,5,6]. After prostatectomy, follow-up is important for detecting recurrence and facilitating timely salvage treatment. Serum prostate-specific antigen (PSA) testing is the principal surveillance method because PSA levels are expected to become undetectable after complete removal of the prostate [7,8,9,10]. Biochemical recurrence (BCR), commonly defined as a postoperative PSA level ≥ 0.2 ng/mL, is associated with subsequent disease progression but does not localize the site of recurrence [11,12,13,14]. Current international recommendations generally discourage routine imaging in patients without a rising PSA and support imaging when recurrence is suspected and the findings are expected to affect treatment planning [15,16]. Multiparametric magnetic resonance imaging (MRI) is particularly useful for evaluating the prostate bed and suspected local recurrence, whereas prostate-specific membrane antigen positron emission tomography (PSMA PET), computed tomography (CT), and bone scintigraphy may provide complementary information regarding nodal, skeletal, or distant disease [17,18,19,20,21,22].

Previous studies have demonstrated variation in imaging after primary PCa treatment and have emphasized that examination-level clinical information is necessary to assess whether post-treatment imaging is appropriate [23,24]. Multiparametric MRI has shown diagnostic utility for evaluating suspected local recurrence in patients with BCR [25]. In addition, greater post-treatment surveillance intensity has been associated with increased use of subsequent procedures and salvage treatments but not with improved overall survival [26]. However, these studies have not specifically quantified repeated postoperative MRI examinations across multiple hospitals within a common healthcare system or examined how institutional differences in MRI utilization change over time after prostatectomy.

In South Korea, patients registered under the cancer-specific copayment reduction program pay 5% of the cost of covered cancer-related medical services for five years from the date of registration [27,28]. National reimbursement criteria separately specify eligible indications and the permitted frequency of MRI examinations after cancer treatment, including postoperative and long-term follow-up imaging [29]. These policies may influence patterns of postoperative MRI utilization. Against this background, this study aimed to evaluate institutional variation and measured clinical factors associated with repeated MRI examinations after prostatectomy and to characterize changes in utilization over postoperative time. Given the potential for postoperative imaging to facilitate the detection and subsequent treatment of recurrence, we additionally explored the association between cumulative MRI use and overall survival as a secondary analysis.

## 2. Materials and Methods

### 2.1. Study Population and Data Sources

This retrospective multicenter cohort study included patients who underwent prostatectomy for PCa between March 2015 and December 2023 at seven different university hospitals. Among 4407 patients who underwent prostatectomy, we excluded patients according to the following criteria: (1) no preoperative PSA level (n = 511); (2) no postoperative PSA measurement available for longitudinal ascertainment of biochemical recurrence (n = 131); (3) an interval of more than one year between the preoperative PSA measurement and prostatectomy (n = 10); and (4) no follow-up after prostatectomy (n = 1). The final analytic cohort comprised 3754 patients (Figure 1).

Deidentified data including patient age, operation date, histopathological results from prostatectomy, and hospital visit dates were retrieved from a clinical data warehouse. The dates and results of prostate MRI and PSA examinations were also collected. Follow-up ended at death for deceased patients and, for other patients, on either the last recorded follow-up or the administrative censoring date of 15 June 2024, whichever occurred earlier.

### 2.2. Definition of Outcomes and Time-Varying Covariates

Preoperative PSA was defined as the measurement obtained before and closest to prostatectomy. Analysis time was defined as the number of days since prostatectomy. A postoperative MRI examination was defined as an MRI examination performed after prostatectomy and within the patient’s follow-up period. BCR was defined as a postoperative PSA level ≥ 0.2 ng/mL, and the BCR date was defined as the date of the first postoperative PSA measurement meeting this threshold. BCR was modeled as a persistent time-updated covariate from that date onward. The temporal relationships among prostatectomy, PSA measurements, MRI examinations, and the end of follow-up are illustrated in Figure 2.

Continuous postoperative PSA was evaluated only in sensitivity analyses using information available at the beginning of each observation interval. Two time-updated measures were considered: (1) the maximum PSA measured since prostatectomy before the first MRI examination or since the preceding MRI examination thereafter and (2) the most recently available PSA measurement. These measures were updated at the beginning of every interval, including intervals without an MRI event. PSA measurements performed on the same date as an MRI examination were not used for that examination because the temporal order could not be determined.

Pathological grade was categorized according to the World Health Organization/International Society of Urological Pathology Grade Group system. Clinically significant PCa was defined as grade group ≥ 2, and grade group was dichotomized as 1 versus ≥2 for the regression analyses [30]. Pathological T stage was categorized as organ-confined disease (T1–T2) versus extraprostatic disease (≥T3) [31].

Postoperative time was measured from prostatectomy, which provided a uniform clinical index date for all patients. PCa registration dates were available, but they were not used to define insurance exposure because re-registration, extension of eligibility, and examination-level reimbursement status could not be reliably determined.

### 2.3. Statistical Analysis

Patient characteristics were compared across hospitals using chi-square tests for categorical variables and Kruskal–Wallis tests for continuous variables. These were omnibus comparisons across all seven hospitals, without pairwise post hoc testing.

A Cox proportional hazards model using an Andersen–Gill counting-process formulation was applied to repeated postoperative MRI examinations, using total time since prostatectomy as the analysis time. All eligible MRI examinations were included as events, without stratification according to event order. Robust sandwich variance estimates were clustered by patient to account for within-patient correlation [32]. The model included hospital, preoperative PSA, time-updated BCR, grade group, pathological T stage, and age, with Hospital 1 as the reference institution.

Preoperative and postoperative PSA were analyzed per 5-ng/mL and 1-ng/mL increases, respectively. Effect estimates were reported to three decimal places for preoperative PSA and four decimal places for postoperative PSA to avoid masking small associations through rounding.

A complementary Poisson regression model was fitted to interval-level MRI counts, using the logarithm of person-time as an offset and robust standard errors clustered by patient. Time since prostatectomy was modeled using a natural cubic spline with four degrees of freedom rather than a prespecified five-year cutoff. Annual crude MRI rates were calculated per 100 person-years, and the adjusted temporal pattern was estimated from the fitted Poisson model.

The proportional-hazards assumption was assessed using scaled Schoenfeld residuals. Because the assumption was violated for hospital, an extended Cox model included interactions between hospital and log(1 + postoperative time). A joint Wald test was used to evaluate the hospital-by-time interaction terms, and time-specific hospital hazard ratios (HRs) were estimated at postoperative years 1, 3, and 5. Hospital-specific utilization was further evaluated using crude and standardized MRI rates. The robustness of the institutional associations was assessed using leave-one-hospital-out sensitivity analyses. Hospital 1 remained the reference institution when Hospitals 2–7 were excluded, whereas Hospital 6 was used as the reference when Hospital 1 was excluded because it had the largest cohort among the remaining institutions. Hospital effects were interpreted as institution-level variation rather than as measures of individual physician practice. Further details of the longitudinal data structure, temporal analyses, model diagnostics, standardization, and sensitivity analyses are provided in the Supplementary Methods.

Overall survival was evaluated as an exploratory secondary outcome using a separate counting-process Cox model, with cumulative MRI use included as a time-updated covariate and robust variance estimates clustered by patient. The model was adjusted for hospital, preoperative PSA, time-updated BCR, grade group, pathological T stage, and age.

A two-sided *p* value < 0.05 was considered to indicate statistical significance. All the statistical analyses were performed using R software, version 4.4.2 (R Foundation for Statistical Computing, Vienna, Austria).

This study was approved by the institutional review board of our institution. The approval covered the integrated multicenter dataset provided through the clinical data warehouse, including deidentified data from all seven participating hospitals. The requirement for informed consent was waived because of the retrospective nature of this study and the use of deidentified clinical data.

## 3. Results

### 3.1. Subjects

The characteristics of the included patients (n = 3754) are summarized in Table 1. The median age of the patients was 70 years (interquartile range [IQR], 65–75 years). The numbers of patients treated at the different hospitals reflect the varying sizes of the institutions; more than half of the patients in the cohort (55.8%, 2093/3754) were treated at Hospital 1. Grade group 1 PCa was identified in 12.3% of patients (462/3754). The majority of patients (61.2%, 2296/3754) were confirmed to have organ-confined PCa, with 0.6% (23/3754) at the T1 stage and 60.7% (2273/3754) at the T2 stage. BCR was observed in 10.4% (391/3754) of patients, and the mortality rate was 2.6%. All evaluated patient characteristics except death differed among hospitals (all *p* values < 0.001). The median follow-up interval for all patients in the cohort was 936 (IQR, 432–1687) days. The institutional distribution and reasons for exclusion are presented in Supplementary Table S1, and the available characteristics of included and excluded patients are compared in Supplementary Table S2.

### 3.2. Hospital-Specific MRI Utilization

A total of 2027 MRI examinations were performed during follow-up among the 3754 included patients, corresponding to an average of 0.54 MRI examinations per patient. The crude MRI rate ranged from 3.67 examinations per 100 person-years at Hospital 1 to 87.58 at Hospital 2, and the corresponding standardized rates ranged from 3.69 to 84.30 per 100 person-years (Figure 3A, Supplementary Table S3). The proportion of patients who underwent no postoperative MRI ranged from 14.1% to 89.7%, whereas the proportion who underwent two or more examinations ranged from 1.2% to 58.4% (Figure 3B).

### 3.3. Factors Associated with Repeated Postoperative MRI Examinations

In the primary counting-process Cox model, institution was the factor most strongly associated with repeated MRI use. Compared with Hospital 1, repeated MRI use was significantly higher at all other institutions (HRs averaged over follow-up, 4.083–23.136; all *p* < 0.001) (Figure 4A, Table 2). BCR was associated with increased MRI use after prostatectomy (HR, 1.565; 95% confidence interval [CI], 1.371–1.786; *p* < 0.001). Older age was associated with less frequent MRI use (HR per year, 0.984; 95% CI, 0.978–0.990; *p* < 0.001) (Figure 4B). Higher preoperative PSA was associated with a small reduction in MRI use (HR per 5-ng/mL increase, 0.988; 95% CI, 0.978–0.998; *p* = 0.023). Grade group and pathological T stage were not significantly associated with repeated MRI use. The complementary Poisson regression yielded similar results.

The proportional-hazards assumption was violated for hospital (*p* < 0.001) but not for the other covariates (Supplementary Table S4). The hospital-by-time interaction terms were jointly significant (Wald χ^2^ = 87.23; *p* < 0.001), indicating that the magnitude of the institutional associations changed during follow-up (Supplementary Figure S1). Relative to Hospital 1, the time-specific HR for Hospital 2 decreased from 24.76 in postoperative year 1 to 17.11 in year 5. In contrast, the corresponding HRs increased from 3.13 to 9.53 for Hospital 3, from 14.15 to 33.12 for Hospital 4, from 11.37 to 27.37 for Hospital 5, from 5.12 to 7.36 for Hospital 6, and from 2.43 to 9.31 for Hospital 7 (Supplementary Table S5). The hospital-by-time interactions were statistically significant for Hospitals 3, 4, 5, and 7 but not for Hospitals 2 and 6. Accordingly, the HRs from the primary model were interpreted as average institutional effects over follow-up.

### 3.4. Temporal Pattern of MRI Utilization

MRI utilization changed over postoperative time. The annual crude MRI rate increased from 16.10 examinations per 100 person-years in postoperative year 1 to 24.28 in year 2 and subsequently declined. The largest year-to-year reduction was observed between postoperative years 5 and 6, when the rate decreased from 18.08 to 5.94 examinations per 100 person-years (Figure 5A, Supplementary Table S6). The complementary Poisson spline analysis also demonstrated a nonlinear temporal pattern in MRI utilization over postoperative time (Figure 5B).

### 3.5. Sensitivity and Exploratory Analyses

In sensitivity analyses, neither interval-maximum postoperative PSA (HR per 1-ng/mL increase, 0.9998; 95% CI, 0.9988–1.0009; *p* = 0.739) nor latest-available postoperative PSA (HR per 1-ng/mL increase, 1.0000; 95% CI, 0.9996–1.0003; *p* = 0.838) was significantly associated with repeated MRI use. The postoperative PSA sensitivity analyses included 3722 patients and 1894 MRI examinations for which the required time-updated PSA information was available. The leave-one-hospital-out sensitivity analyses continued to show substantial institutional variation when each nonreference hospital was excluded while retaining Hospital 1 as the reference. After Hospital 1 was excluded, substantial variation also remained among Hospitals 2–7 (overall robust Wald χ^2^ = 759.03; df = 5; *p* < 0.001), with hospital-specific HRs ranging from 0.703 to 4.080 relative to Hospital 6 (Supplementary Table S7).

Cumulative MRI examinations were not significantly associated with overall survival (HR, 0.782; 95% CI, 0.564–1.086; *p* = 0.142) (Supplementary Table S8). Older age, BCR, and higher preoperative PSA were significantly associated with an increased risk of death. Hospital was not significantly associated with mortality in the overall joint test (Wald χ^2^ = 7.86; df = 6; *p* = 0.249), although the individual comparison between Hospital 3 and Hospital 1 reached nominal statistical significance (HR, 0.231; 95% CI, 0.055–0.969; *p* = 0.045). Grade group and pathological T stage were not significantly associated with overall survival.

## 4. Discussion

In this multicenter study of 3754 patients treated at seven hospitals, 2027 postoperative MRI examinations were identified during follow-up. Postoperative MRI utilization varied markedly across institutions, and these differences persisted after adjustment for measured clinical factors. BCR was associated with increased MRI use, whereas older age and higher preoperative PSA showed small inverse associations. Grade group and pathological T stage were not significantly associated with repeated MRI use, nor was either time-updated postoperative PSA measure. MRI utilization also changed nonlinearly over postoperative time, with the largest year-to-year reduction occurring between postoperative years 5 and 6. Finally, cumulative MRI use was not significantly associated with overall survival in the exploratory secondary analysis. Collectively, these findings demonstrate substantial institutional and temporal variation in postoperative MRI utilization that was not fully explained by the measured clinical factors.

The most striking finding was the magnitude of institutional variation in postoperative MRI utilization, with adjusted HRs reaching approximately 23 relative to Hospital 1. These associations remained substantial in the complementary Poisson and leave-one-hospital-out analyses, although their magnitude changed over postoperative time; accordingly, the HRs from the primary model represent average institutional effects over follow-up. Differences in institutional practice patterns, implementation of surveillance guidelines, follow-up practices, and MRI availability may have contributed to the observed variation. Previous studies described variation in imaging after primary prostate cancer treatment and evaluated whether post-treatment imaging was performed in accordance with appropriateness criteria [23,24]. Our study extends this work by quantifying repeated MRI use across seven hospitals within a common healthcare system and by demonstrating that the magnitude of the institutional contrasts changed over postoperative time. These findings support the development of clearer evidence-based criteria and more standardized postoperative imaging pathways.

The association between BCR and more frequent postoperative MRI examinations is clinically plausible because a rising PSA commonly prompts imaging to localize suspected recurrence. A previous Korean study reported a low diagnostic yield when multiparametric MRI was applied broadly to patients with BCR and suggested more selective use according to PSA level [25]. Together, these findings suggest that although BCR is an important trigger for postoperative MRI, more specific evidence-based criteria may help identify patients most likely to benefit from imaging.

MRI utilization increased during the early postoperative period and subsequently declined, with the largest observed year-to-year reduction occurring between postoperative years 5 and 6. This timing coincides with the usual five-year period of the Korean cancer-specific copayment reduction program and therefore provides a potential policy context for the observed pattern [27,28,29]. A Korean interrupted time-series study using national claims data found that restricting reimbursement for F-18 fluorodeoxyglucose positron emission tomography in patients with breast cancer was followed by an immediate decrease in its use and increases in the use of some alternative imaging modalities [33]. The decline may also reflect concurrent changes in clinical need, follow-up intensity, transfer of care, or the use of alternative imaging modalities. Future studies incorporating claims data and examination-level reimbursement information may help clarify the relative contributions of these clinical and policy-related factors.

Cumulative MRI use was not significantly associated with overall survival. This finding parallels the AFT-30 study, in which greater post-treatment PSA surveillance intensity was associated with more subsequent procedures and salvage treatments but not with improved overall survival [26]. Although that study evaluated PSA surveillance rather than MRI utilization, both findings indicate that an association between surveillance intensity and short-term overall survival is not readily demonstrated in observational data. In the present study, older age, BCR, and higher preoperative PSA were associated with an increased risk of death, consistent with findings from previous studies [11,34,35,36,37]. Longer follow-up with more deaths and detailed information on comorbidities, disease progression, and subsequent treatments will be needed to better characterize the relationship between postoperative imaging and clinical outcomes.

A strength of this study was the inclusion of longitudinal PSA and MRI data from seven hospitals through a centralized clinical data warehouse. Such detailed information on follow-up examinations, including PSA and MRI, is difficult to obtain from public datasets. The longitudinal data allowed BCR status to be updated over follow-up and all postoperative MRI examinations to be included in a repeated-event analysis. The robustness of the findings was further supported by the consistency between the Andersen–Gill and Poisson models, assessment of the proportional-hazards assumption, hospital-by-time analyses, leave-one-hospital-out analyses, and sensitivity analyses using two time-updated postoperative PSA measures.

This study has several limitations. First, physician-level practice patterns, MRI indications, symptoms, MRI findings, and subsequent treatment decisions were unavailable; therefore, the appropriateness of individual examinations and the mechanisms underlying the institutional variation could not be determined. Information on CT, PSMA PET/CT, bone scintigraphy, outside imaging, institutional MRI capacity, and waiting times was also incomplete, and MRI utilization should not be interpreted as total imaging intensity. Second, individual eligibility for the cancer-specific copayment reduction program, re-registration or extension of coverage, examination-level reimbursement, and out-of-pocket costs could not be reliably determined; therefore, temporal changes in MRI utilization cannot be attributed causally to insurance coverage. Third, institutional differences in exclusion rates, PSA testing frequency, follow-up, referral patterns, case mix, and completeness of outside records may have contributed to the observed variation despite multivariable adjustment and sensitivity analyses. Finally, the exploratory survival analysis was limited by the small number of deaths, relatively short follow-up period, use of all-cause mortality, and unavailable information on comorbidities and subsequent treatments.

In conclusion, MRI utilization after prostatectomy varied substantially across institutions and over postoperative time, with a marked decline after postoperative year 5. Although MRI examinations were more frequent after BCR, the large institutional differences were not fully explained by the measured clinical factors. These findings highlight the need for more standardized and evidence-based criteria for MRI use after prostatectomy.

## Figures and Tables

**Figure 1:**
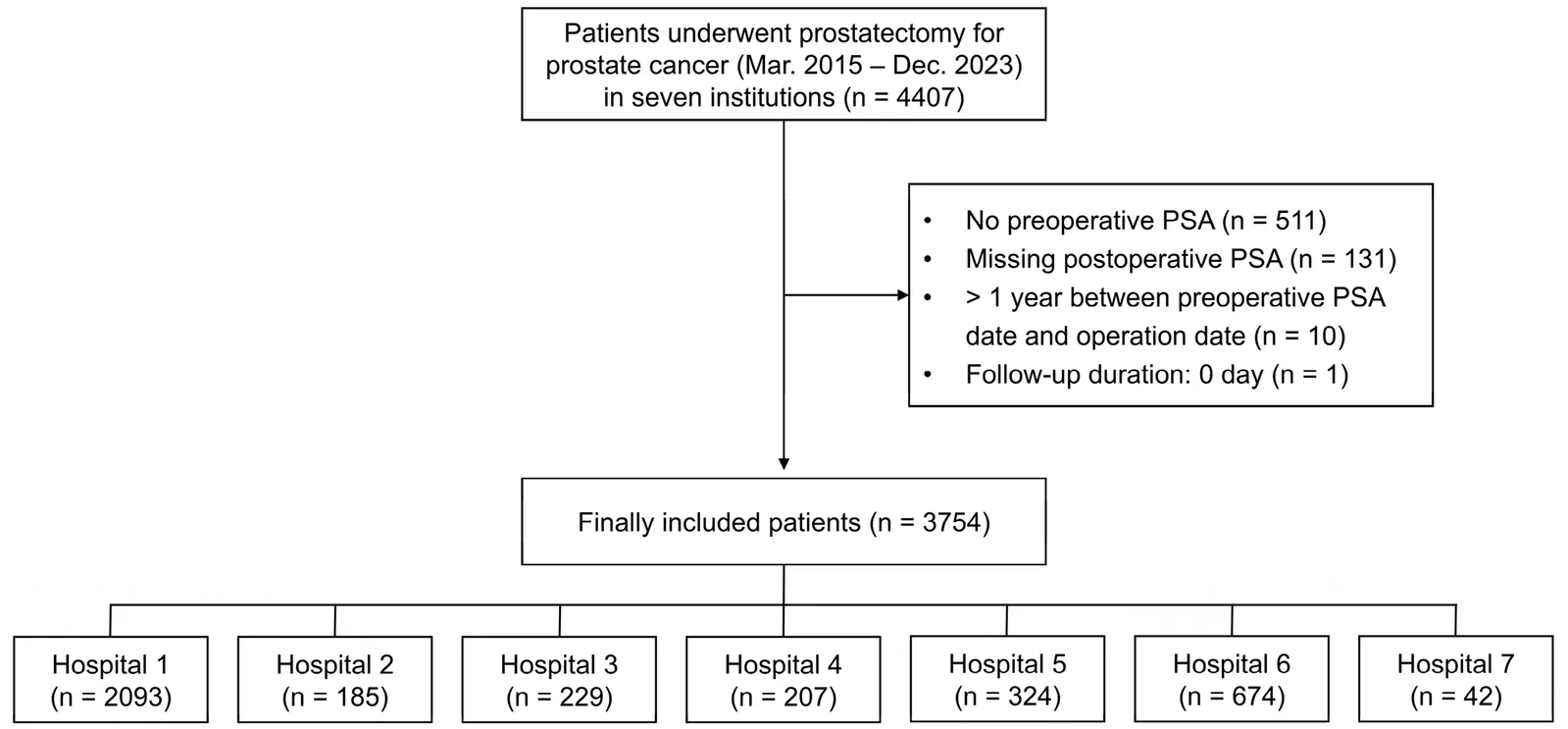
Flow chart for patient inclusion. PSA, prostate-specific antigen.

**Figure 2:**
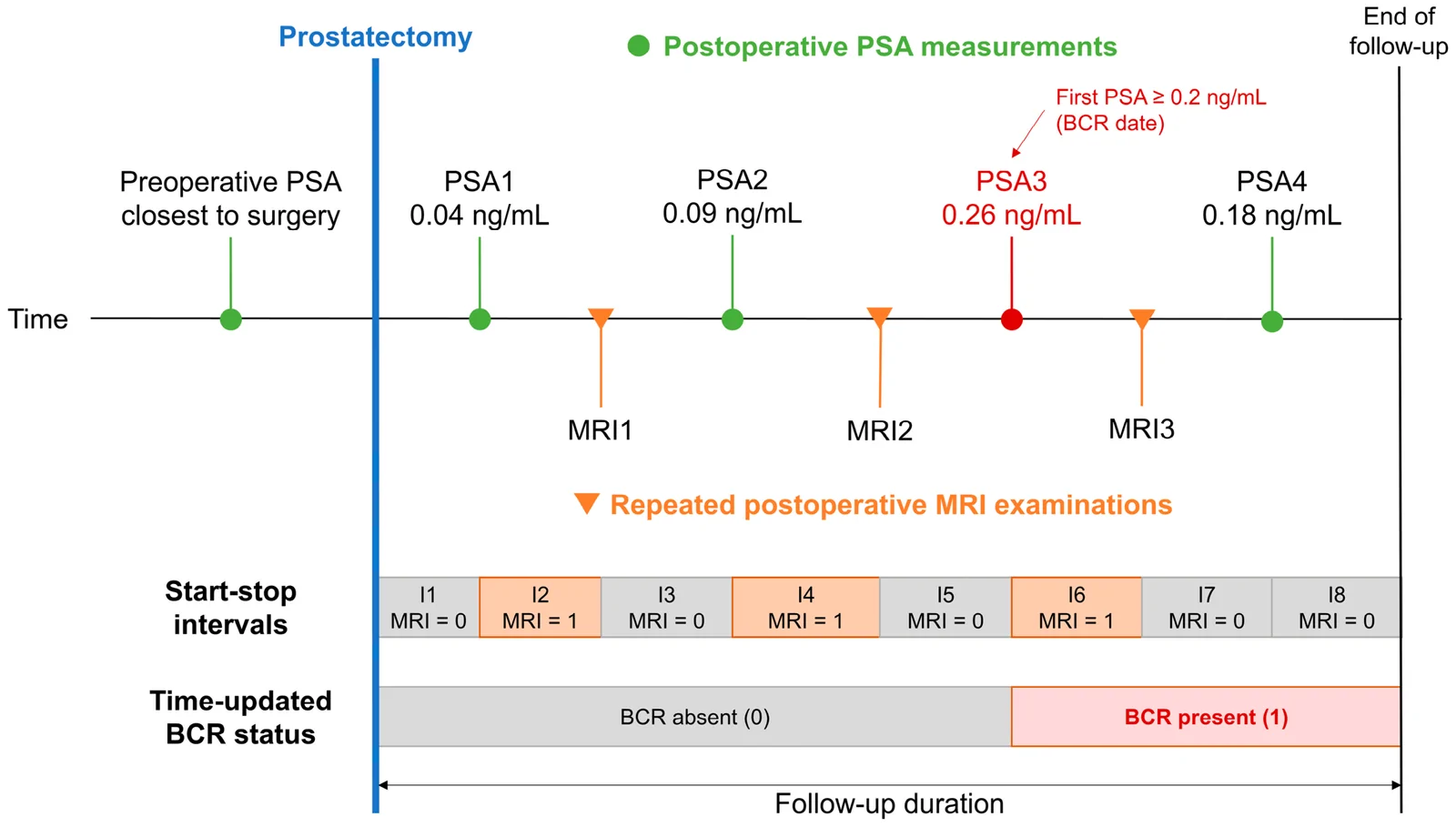
Illustrative longitudinal data structure. Follow-up began at prostatectomy and was divided into start–stop intervals according to the dates of postoperative PSA measurements, MRI examinations, changes in biochemical recurrence status, and the end of follow-up. An MRI examination occurring at the end of an interval was coded as an event for that interval. Biochemical recurrence status changed from absent to present on the date of the first postoperative PSA measurement ≥ 0.2 ng/mL and remained present thereafter. Continuous postoperative PSA was evaluated only in sensitivity analyses using PSA information available at the beginning of each interval. BCR, biochemical recurrence; MRI, magnetic resonance imaging; PSA, prostate-specific antigen.

**Figure 3:**
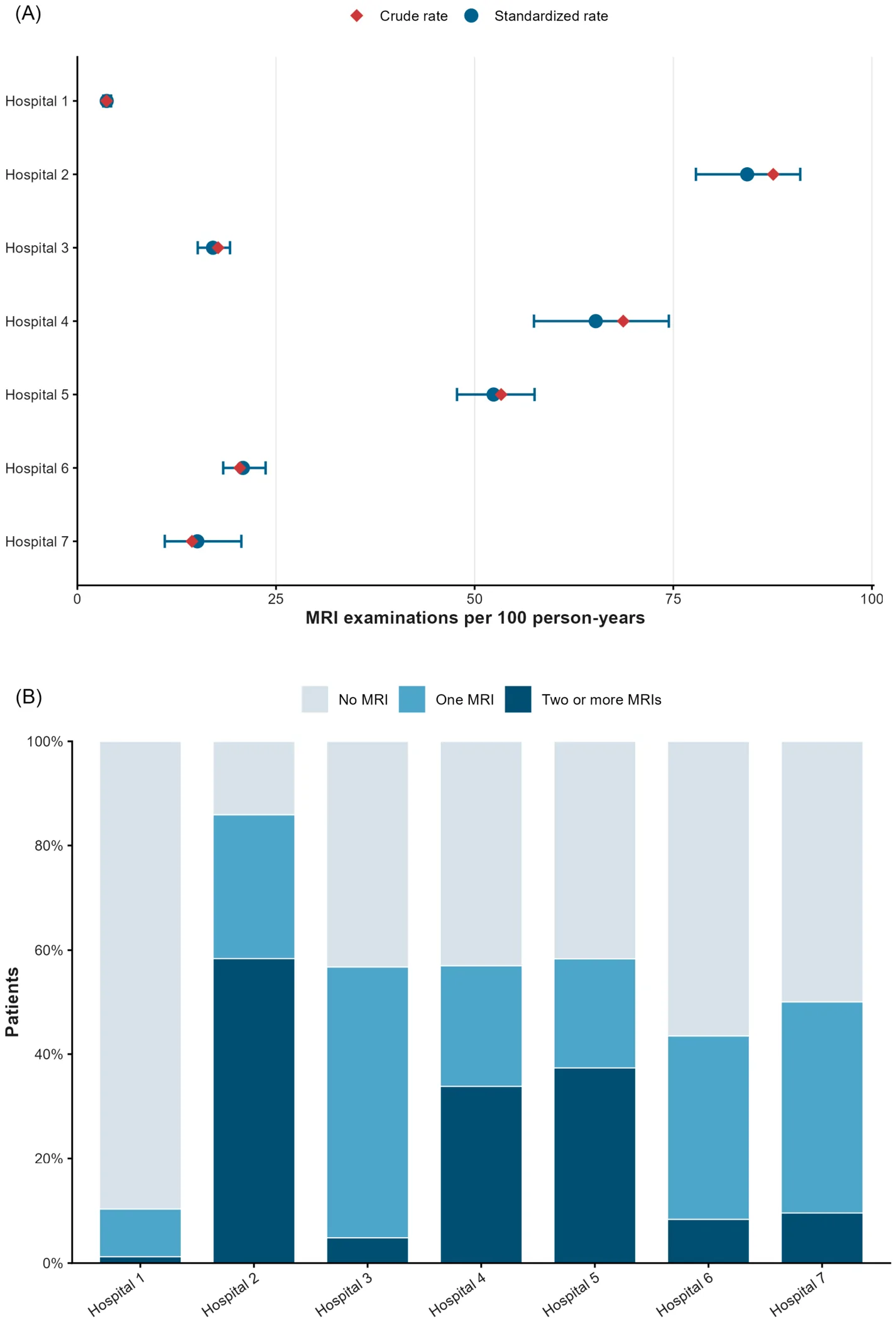
Institutional variation in postoperative MRI utilization. Crude and standardized rates of postoperative MRI examinations varied markedly across hospitals (**A**). Red diamonds indicate crude rates, and blue circles with horizontal error bars indicate standardized rates and their 95% confidence intervals. Standardized rates were estimated from the complementary Poisson model while retaining the observed distributions of postoperative time, clinical covariates, and person-time. The proportions of patients undergoing no MRI, one MRI, or two or more MRI examinations during follow-up also varied across hospitals (**B**). MRI, magnetic resonance imaging.

**Figure 4:**
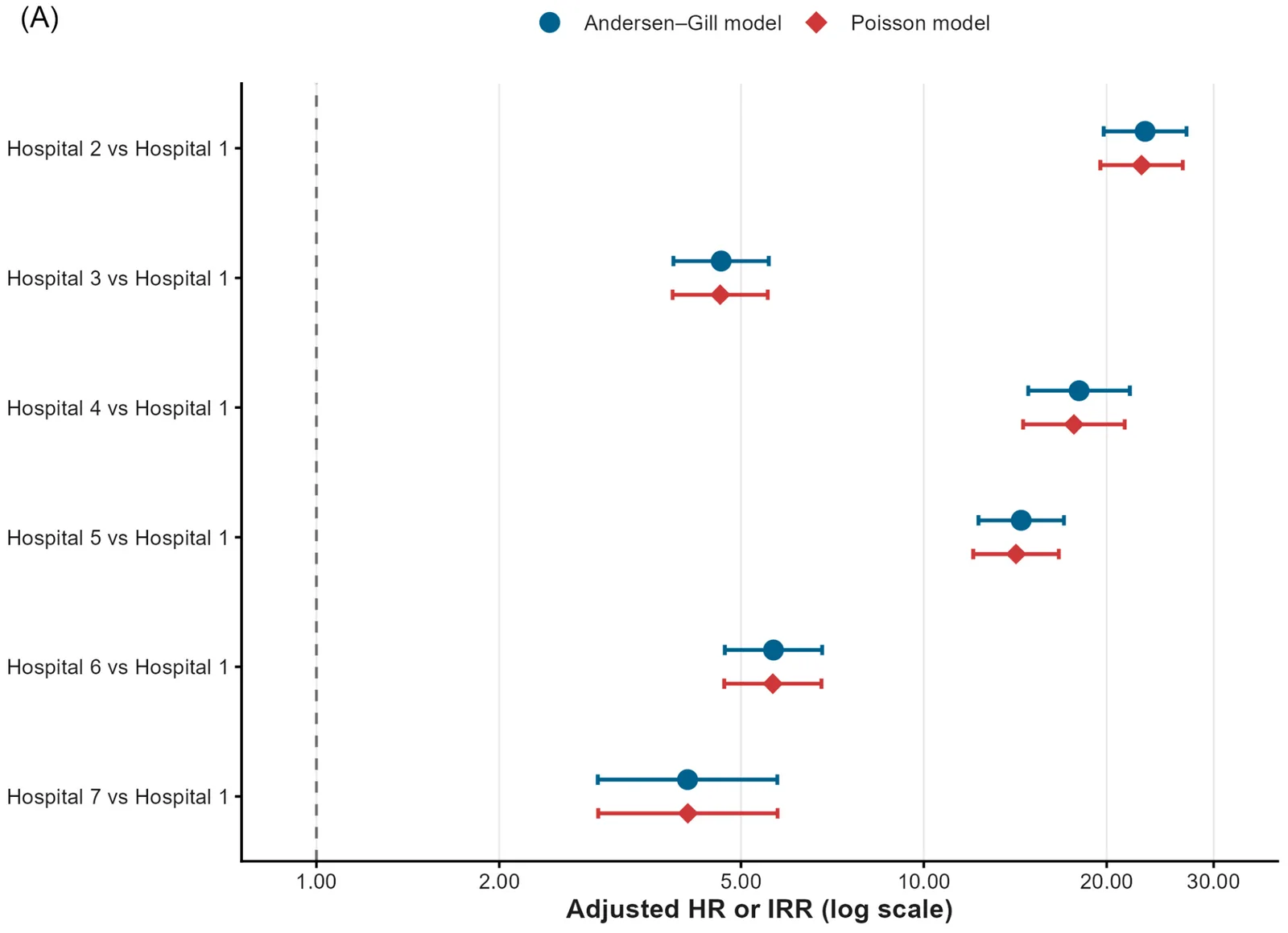

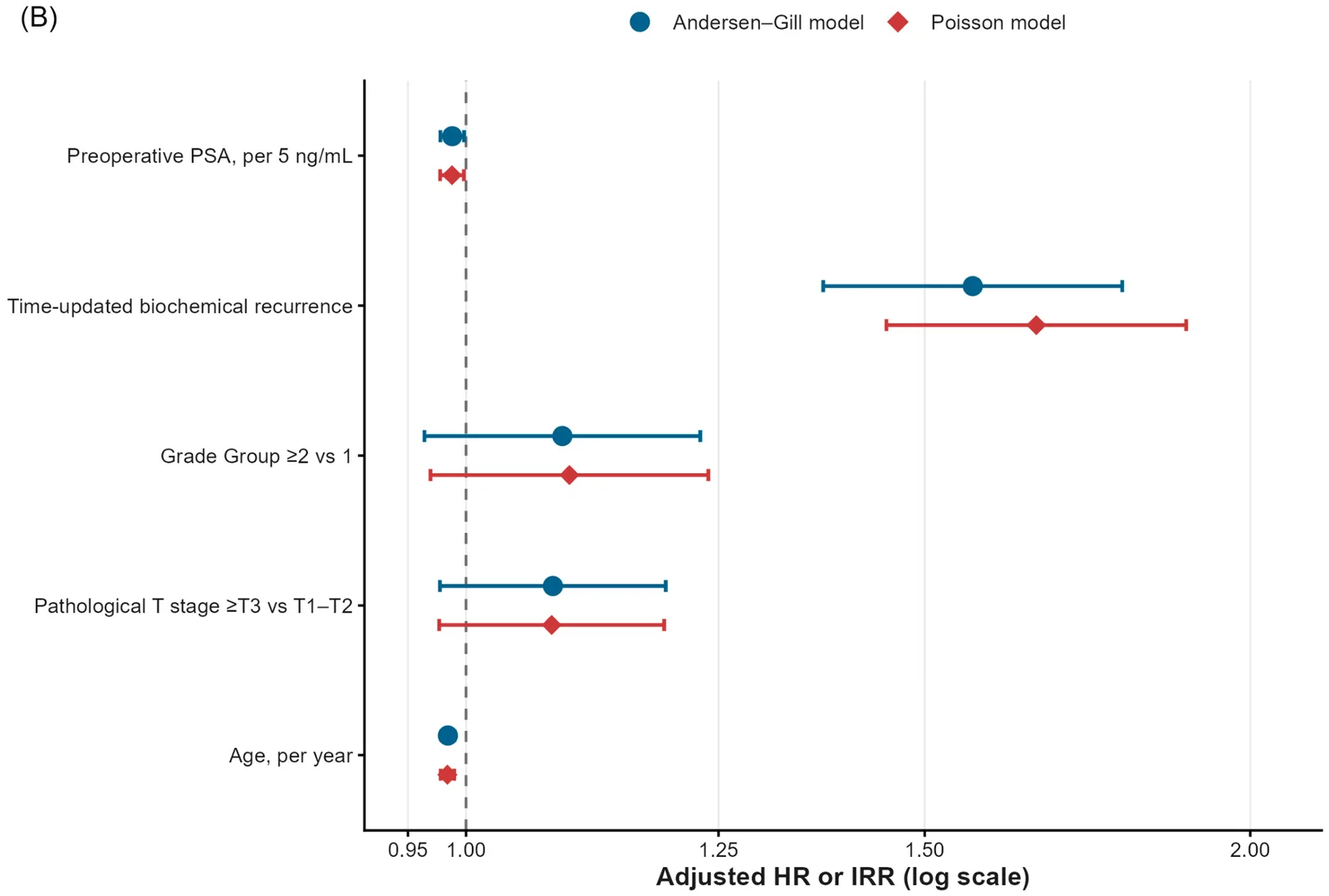
Adjusted associations with repeated postoperative MRI use in the Andersen–Gill and Poisson models. Adjusted hospital-specific estimates relative to Hospital 1 are shown for the Andersen–Gill and complementary Poisson models (**A**). Blue circles indicate hazard ratios from the Andersen–Gill model, and red diamonds indicate incidence rate ratios from the Poisson model; horizontal error bars indicate 95% confidence intervals. Adjusted estimates for the measured clinical and pathological factors are shown for both models (**B**) using the same symbols. The vertical dashed line indicates an HR or IRR of 1. HR, hazard ratio; IRR, incidence rate ratio; PSA, prostate-specific antigen.

**Figure 5:**
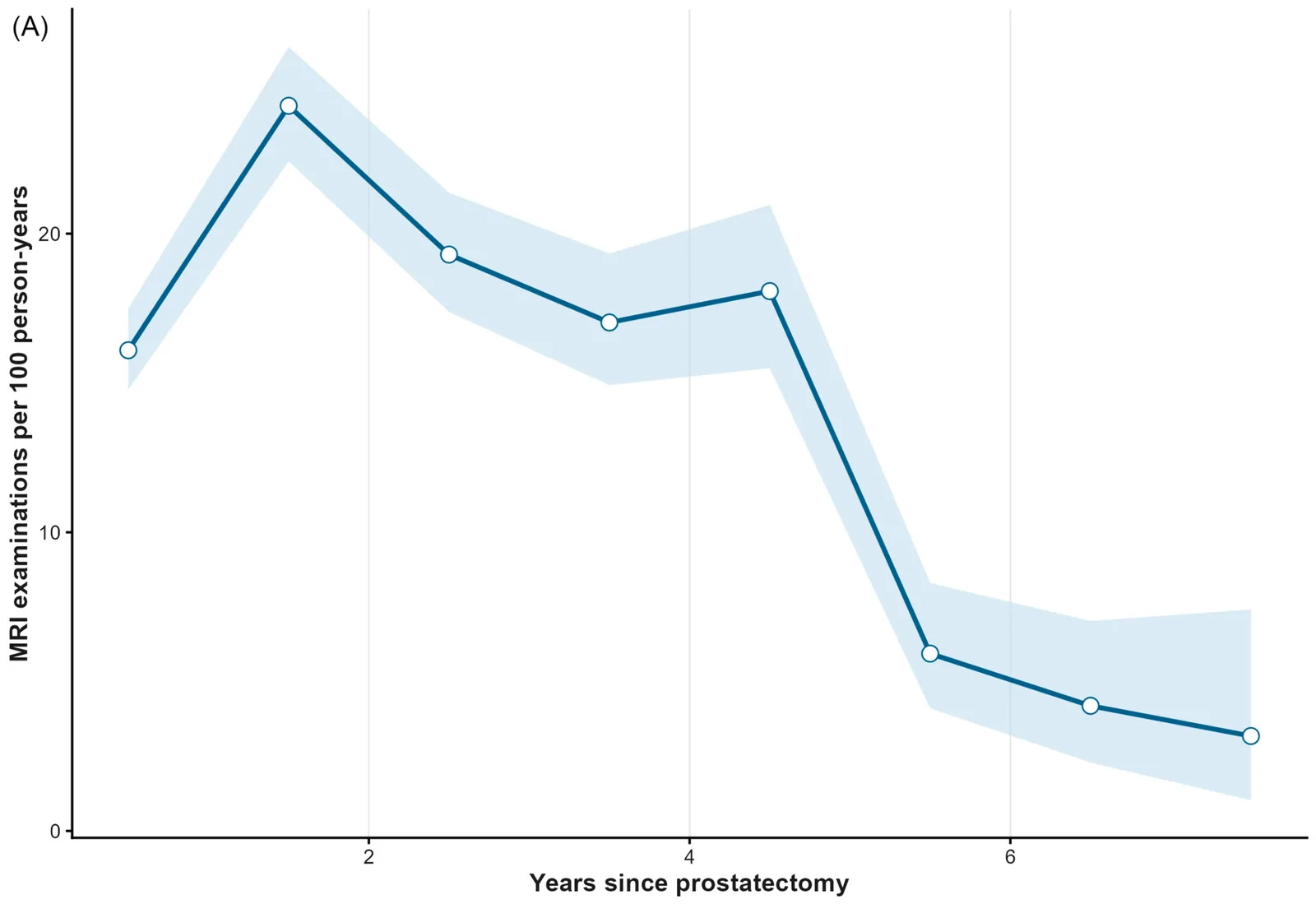

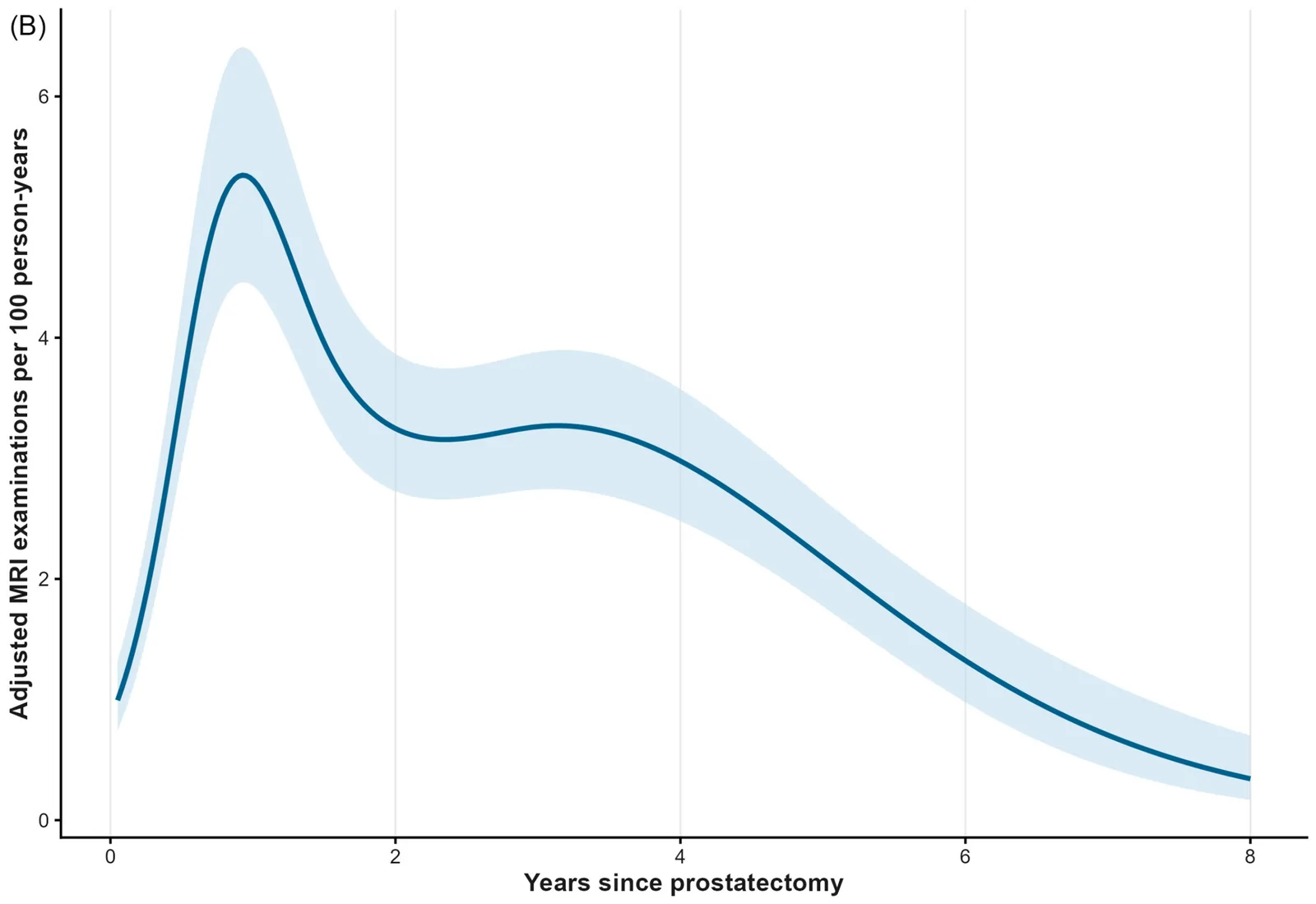
Temporal pattern of postoperative MRI utilization. Annual crude MRI rates varied across postoperative years (**A**). Points indicate rates per 100 person-years, and error bars indicate exact Poisson 95% confidence intervals. The adjusted temporal utilization curve estimated from the complementary Poisson spline model demonstrates the nonlinear change in MRI utilization over postoperative time (**B**). The solid line indicates the estimated rate for the reference clinical profile, and the shaded area indicates the 95% confidence interval. MRI, magnetic resonance imaging.

**Table 1:** Patient characteristics according to hospital.

Variable	All Patients	Hospital							*p* Value
		1	2	3	4	5	6	7	
Number of patients (n)	3754	2093	185	229	207	324	674	42	-
Age (years)	70 (65–75)	69 (64–74)	71 (65–75)	68 (64–74)	72 (67–77)	73 (67–77)	70 (65–74)	70 (66–73)	<0.001
Preoperative PSA value (ng/mL)	7.8 (5.3–13.7)	7.6 (5.1–13.2)	7.6 (5.4–13.6)	7.1 (5.1–11.7)	9.2 (6.0–19.3)	8.1 (5.2–13.0)	8.9 (6.0–16.2)	7.7 (4.5–12.0)	<0.001
Follow-up interval (days)	936 (432–1687)	1021 (481–1759)	986 (374–1392)	1336 (547–1846)	582 (294–1076)	768 (370–1395)	822 (409–1615)	1331 (798–2408)	<0.001
Grade group									<0.001
1	462 (12.3)	161 (7.7)	28 (15.1)	44 (19.2)	14 (6.8)	49 (15.1)	160 (23.7)	6 (14.3)	
2	1485 (39.6)	843 (40.3)	80 (43.2)	84 (36.7)	89 (43.0)	175 (54.0)	206 (30.6)	8 (19.0)	
3	1082 (28.8)	704 (33.6)	34 (18.4)	61 (26.6)	59 (28.5)	56 (17.3)	158 (23.4)	10 (23.8)	
4	313 (8.3)	198 (9.5)	20 (10.8)	20 (8.7)	6 (2.9)	15 (4.6)	43 (6.4)	11 (26.2)	
5	412 (11.0)	187 (8.9)	23 (12.4)	20 (8.7)	39 (18.8)	29 (9.0)	107 (15.9)	7 (16.7)	
Clinically significant cancer	3292 (87.7)	1932 (92.3)	157 (84.9)	185 (80.8)	193 (93.2)	275 (84.9)	514 (76.3)	36 (85.7)	<0.001
Pathological T stage									<0.001
T1	23 (0.6)	3 (0.1)	0 (0)	3 (1.3)	0 (0)	5 (1.5)	3 (0.4)	9 (21.4)	
T2	2273 (60.5)	1240 (59.2)	127 (68.6)	152 (66.4)	103 (49.8)	238 (73.5)	388 (57.6)	25 (59.5)	
T3a	902 (24.0)	521 (24.9)	41 (22.2)	50 (21.8)	58 (28.0)	54 (16.7)	173 (25.7)	5 (11.9)	
T3b	540 (14.4)	320 (15.3)	16 (8.6)	23 (10.0)	45 (21.7)	26 (8.0)	107 (15.9)	3 (7.1)	
T4	16 (0.4)	9 (0.4)	1 (0.5)	1 (0.4)	1 (0.8)	1 (0.3)	3 (0.4)	0 (0)	
Organ-confined cancer	2296 (61.2)	1243 (59.4)	127 (68.6)	155 (67.7)	103 (49.8)	243 (75.0)	391 (58.0)	34 (81.0)	<0.001
Biochemical recurrence	391 (10.4)	131 (6.3)	31 (16.8)	42 (18.3)	46 (22.2)	55 (17.0)	78 (11.6)	8 (19.0)	<0.001
Death	98 (2.6)	70 (3.3)	4 (2.2)	2 (0.9)	4 (1.9)	7 (2.2)	10 (1.5)	1 (2.4)	0.079

Data are presented as numbers (percentages) or medians (interquartile ranges). Percentages may not sum to 100% because of rounding. *p* values were calculated using Kruskal –Wallis tests for continuous values or chi-square tests for categorical values among hospitals. Clinically significant cancer was defined as grade group ≥ 2, and organ-confined cancer was defined as pathological stage T1 or T2. PSA, prostate-specific antigen.

**Table 2:** Factors associated with repeated postoperative MRI examinations.

Variable	Andersen–Gill Model		Poisson Model	
	HR (95% CI)	*p* Value	IRR (95% CI)	*p* Value
Hospital 1	Reference	—	Reference	—
Hospital 2	23.136 (19.771–27.073)	<0.001	22.827 (19.523–26.690)	<0.001
Hospital 3	4.637 (3.870–5.556)	<0.001	4.621 (3.858–5.534)	<0.001
Hospital 4	18.012 (14.856–21.839)	<0.001	17.666 (14.571–21.418)	<0.001
Hospital 5	14.465 (12.299–17.012)	<0.001	14.185 (12.061–16.682)	<0.001
Hospital 6	5.655 (4.703–6.800)	<0.001	5.641 (4.692–6.783)	<0.001
Hospital 7	4.083 (2.906–5.737)	<0.001	4.088 (2.910–5.743)	<0.001
Preoperative PSA, per 5 ng/mL	0.988 (0.978–0.998)	0.023	0.988 (0.977–0.998)	0.020
Biochemical recurrence	1.565 (1.371–1.786)	<0.001	1.655 (1.450–1.890)	<0.001
Grade group ≥ 2 vs. 1	1.089 (0.964–1.230)	0.171	1.096 (0.969–1.239)	0.145
Pathological T stage ≥ T3 vs. T1–T2	1.080 (0.977–1.193)	0.132	1.079 (0.977–1.191)	0.136
Age, per year	0.984 (0.978–0.990)	<0.001	0.984 (0.978–0.990)	<0.001

Hospital-specific HRs represent average institutional effects over follow-up because the proportional-hazards assumption was violated for hospital. Biochemical recurrence was modeled as a time-updated covariate. The Poisson model included a natural cubic spline for postoperative time, an offset for person-time, and patient-clustered robust standard errors. CI, confidence interval; HR, hazard ratio; IRR, incidence rate ratio; PSA, prostate-specific antigen.

## Data Availability

The data presented in this study are not publicly available due to patient privacy and institutional restrictions. Deidentified data may be available from the corresponding author upon reasonable request and with approval from the relevant institutional review boards and participating institutions.

## Supplementary Materials

The following supporting information can be downloaded at https://www.mdpi.com/article/10.3390/healthcare14193131/s1.

## References

[1] Bray F., Laversanne M., Sung H., Ferlay J., Siegel R.L., Soerjomataram I., Jemal A. (2024). Global cancer statistics 2022: GLOBOCAN estimates of incidence and mortality worldwide for 36 cancers in 185 countries. CA Cancer J. Clin..

[2] Siegel D.A., O’Neil M.E., Richards T.B., Dowling N.F., Weir H.K. (2020). Prostate Cancer Incidence and Survival, by Stage and Race/Ethnicity—United States, 2001–2017. Morb. Mortal. Wkly. Rep..

[3] Loeb S., Bjurlin M.A., Nicholson J., Tammela T.L., Penson D.F., Carter H.B., Carroll P., Etzioni R. (2014). Overdiagnosis and overtreatment of prostate cancer. Eur. Urol..

[4] Bangma C.H., Roemeling S., Schroder F.H. (2007). Overdiagnosis and overtreatment of early detected prostate cancer. World J. Urol..

[5] Klotz L. (2013). Prostate cancer overdiagnosis and overtreatment. Curr. Opin. Endocrinol. Diabetes. Obes..

[6] Walsh P.C., DeWeese T.L., Eisenberger M.A. (2007). Clinical practice. Localized prostate cancer. N. Engl. J. Med..

[7] Stephenson A.J., Scardino P.T., Eastham J.A., Bianco F.J., Dotan Z.A., Fearn P.A., Kattan M.W. (2006). Preoperative nomogram predicting the 10-year probability of prostate cancer recurrence after radical prostatectomy. J. Natl. Cancer. Inst..

[8] Stephenson A.J., Scardino P.T., Eastham J.A., Bianco F.J., Dotan Z.A., DiBlasio C.J., Reuther A., Klein E.A., Kattan M.W. (2005). Postoperative nomogram predicting the 10-year probability of prostate cancer recurrence after radical prostatectomy. J. Clin. Oncol..

[9] Brockman J.A., Alanee S., Vickers A.J., Scardino P.T., Wood D.P., Kibel A.S., Lin D.W., Bianco F.J., Rabah D.M., Klein E.A. (2015). Nomogram Predicting Prostate Cancer-specific Mortality for Men with Biochemical Recurrence After Radical Prostatectomy. Eur. Urol..

[10] Eastham J.A., Auffenberg G.B., Barocas D.A., Chou R., Crispino T., Davis J.W., Eggener S., Horwitz E.M., Kane C.J., Kirkby E. (2022). Clinically Localized Prostate Cancer: AUA/ASTRO Guideline, Part II: Principles of Active Surveillance, Principles of Surgery, and Follow-Up. J. Urol..

[11] Van den Broeck T., van den Bergh R.C.N., Arfi N., Gross T., Moris L., Briers E., Cumberbatch M., De Santis M., Tilki D., Fanti S. (2019). Prognostic Value of Biochemical Recurrence Following Treatment with Curative Intent for Prostate Cancer: A Systematic Review. Eur. Urol..

[12] Van den Broeck T., van den Bergh R.C.N., Briers E., Cornford P., Cumberbatch M., Tilki D., De Santis M., Fanti S., Fossati N., Gillessen S. (2020). Biochemical Recurrence in Prostate Cancer: The European Association of Urology Prostate Cancer Guidelines Panel Recommendations. Eur. Urol. Focus..

[13] Lowrance W., Dreicer R., Jarrard D.F., Scarpato K.R., Kim S.K., Kirkby E., Buckley D.I., Griffin J.C., Cookson M.S. (2023). Updates to Advanced Prostate Cancer: AUA/SUO Guideline (2023). J. Urol..

[14] Toussi A., Stewart-Merrill S.B., Boorjian S.A., Psutka S.P., Thompson R.H., Frank I., Tollefson M.K., Gettman M.T., Carlson R.E., Rangel L.J. (2016). Standardizing the Definition of Biochemical Recurrence after Radical Prostatectomy-What Prostate Specific Antigen Cut Point Best Predicts a Durable Increase and Subsequent Systemic Progression?. J. Urol..

[15] EAU Guidelines. Edn. Presented at the EAU Annual Congress London 2026. EAU Guidelines Office. http://uroweb.org/guidelines/compilations-of-all-guidelines/.

[16] Morgan T.M., Boorjian S.A., Buyyounouski M.K., Chapin B.F., Chen D.Y.T., Cheng H.H., Chou R., Jacene H.A., Kamran S.C., Kim S.K. (2024). Salvage Therapy for Prostate Cancer: AUA/ASTRO/SUO Guideline Part I: Introduction and Treatment Decision-Making at the Time of Suspected Biochemical Recurrence after Radical Prostatectomy. J. Urol..

[17] Bukavina L., Luckenbaugh A.N., Hofman M.S., Hope T., Kamran S.C., Murphy D.G., Yamoah K., Ost P. (2023). Incorporating Prostate-specific Membrane Antigen Positron Emission Tomography in Management Decisions for Men with Newly Diagnosed or Biochemically Recurrent Prostate Cancer. Eur. Urol..

[18] Jiang Z., Yang T., Xu L. (2024). Head-to-head comparison of prostate-specific membrane antigen positron emission tomography/computed tomography and multiparametric magnetic resonance imaging in the detection of biochemical recurrence of prostate cancer: A systematic review and meta-analysis. Clin. Radiol..

[19] De Visschere P.J.L., Standaert C., Futterer J.J., Villeirs G.M., Panebianco V., Walz J., Maurer T., Hadaschik B.A., Lecouvet F.E., Giannarini G. (2019). A Systematic Review on the Role of Imaging in Early Recurrent Prostate Cancer. Eur. Urol. Oncol..

[20] Panebianco V., Villeirs G., Weinreb J.C., Turkbey B.I., Margolis D.J., Richenberg J., Schoots I.G., Moore C.M., Futterer J., Macura K.J. (2021). Prostate Magnetic Resonance Imaging for Local Recurrence Reporting (PI-RR): International Consensus-based Guidelines on Multiparametric Magnetic Resonance Imaging for Prostate Cancer Recurrence after Radiation Therapy and Radical Prostatectomy. Eur. Urol. Oncol..

[21] Counago F., del Cerro E., Recio M., Diaz A.A., Marcos F.J., Cerezo L., Maldonado A., Rodriguez-Luna J.M., Thuissard I., Martin J.L. (2015). Role of 3T multiparametric magnetic resonance imaging without endorectal coil in the detection of local recurrent prostate cancer after radical prostatectomy: The radiation oncology point of view. Scand. J. Urol..

[22] Bhargava P., Ravizzini G., Chapin B.F., Kundra V. (2020). Imaging Biochemical Recurrence After Prostatectomy: Where Are We Headed?. Am. J. Roentgenol..

[23] McDonald J.S., Carter R.E., Karnes R.J., Port J.D., Kawashima A., Carlson S.K., Bender C.E. (2015). Evaluation of Posttreatment Follow-Up of Patients with Prostate Cancer Relative to the American College of Radiology’s Appropriateness Criteria. Am. J. Roentgenol..

[24] Hussein A.A., Punnen S., Zhao S., Cowan J.E., Leapman M., Tran T.C., Washington S.L., Truesdale M.D., Carroll P.R., Cooperberg M.R. (2015). Current Use of Imaging after Primary Treatment of Prostate Cancer. J. Urol..

[25] Kim M., Hwang S.I., Ahn H., Lee H.J., Byun S.S., Hong S.K., Lee S. (2022). Diagnostic yield of multiparametric MRI for local recurrence at biochemical recurrence after radical prostatectomy. Prostate Int..

[26] Chen R.C., Basak R., Dusetzina S., Usinger D.S., Mohammed Z., Falchook A.D., Schumacher J.R., Francescatti A.B., Cuddy A., Chang G.J. (2024). Posttreatment surveillance intensity and overall survival in prostate cancer survivors (AFT-30). JNCI Cancer Spectr..

[27] National Health Insurance Service Insurance Benefits: Co-Payment for Registered Cancer Patients. https://www.nhis.or.kr/english/wbheaa02600m01.do.

[28] Ministry of Health and Welfare, Republic of Korea Cancer Patient Special Copayment Program: 5% Patient Copayment. Published 24 August 2010. https://www.mohw.go.kr/board.es?act=view&bid=0027&list_no=239755&mid=a10503010100.

[29] Health Insurance Review & Assessment Service National Health Insurance Coverage Criteria for Magnetic Resonance Imaging. Ministry of Health and Welfare Notice No. 2019-77. Published 24 April 2019. https://www.hira.or.kr/rc/insu/insuadtcrtr/InsuAdtCrtrPopup.do?brdScnBltNo=4&isPopupYn=Y&mtgHmeDd=20190424&mtgMtrRegSno=3&sno=1.

[30] Epstein J.I., Egevad L., Amin M.B., Delahunt B., Srigley J.R., Humphrey P.A., Grading Committee (2016). The 2014 International Society of Urological Pathology (ISUP) Consensus Conference on Gleason Grading of Prostatic Carcinoma: Definition of Grading Patterns and Proposal for a New Grading System. Am. J. Surg. Pathol..

[31] (2016). AJCC Cancer Staging Manual.

[32] Lin D.Y., Wei L.J. (1989). The Robust Inference for the Cox Proportional Hazards Model. J. Am. Stat. Assoc..

[33] Kim C., Chun S.Y., Kim S.J., Yang K.H., Baek J.H., Shin J.H., Yoo J.W., Chang Y.W., Kang K.W., Hwang J. (2022). The Impact of the Amendment of the Health Insurance Coverage for F-18 Fluorodeoxyglucose Positron Emission Tomography on the Healthcare Behaviors for Breast Cancer: An Interrupted Time Series Analysis of the Korean National Data from 2013 to 2018. J. Korean Med. Sci..

[34] Siddiqui S.A., Sengupta S., Slezak J.M., Bergstralh E.J., Leibovich B.C., Myers R.P., Zincke H., Blute M.L. (2006). Impact of patient age at treatment on outcome following radical retropubic prostatectomy for prostate cancer. J. Urol..

[35] Buhmeida A., Pyrhonen S., Laato M., Collan Y. (2006). Prognostic factors in prostate cancer. Diagn. Pathol..

[36] Hsu C.Y., Wildhagen M.F., Van Poppel H., Bangma C.H. (2010). Prognostic factors for and outcome of locally advanced prostate cancer after radical prostatectomy. BJU Int..

[37] Liesenfeld L., Kron M., Gschwend J.E., Herkommer K. (2017). Prognostic Factors for Biochemical Recurrence More than 10 Years after Radical Prostatectomy. J. Urol..

