# Supplementary material for: Institutional Variation in MRI Utilization After Prostatectomy for Prostate Cancer: A Multicenter Study
Source: Healthcare (Basel). 2026 Sep 22;14(19):3131. doi: 10.3390/healthcare14193131 (PMC13665149; doi:10.3390/healthcare14193131)

Supplementary Methods

Longitudinal data structure

Follow-up was represented by start–stop intervals using total time since prostatectomy. Interval boundaries were defined by MRI examinations, PSA measurements, changes in BCR status, and the end of follow-up. An MRI examination occurring at the end of an interval was coded as an event. All MRI examinations were analyzed under the Andersen–Gill formulation without stratification by event order, and robust variance estimates were clustered by patient. For the postoperative PSA sensitivity analyses, PSA values were updated at the beginning of each interval, regardless of whether an MRI examination occurred at the end of that interval. Intervals without available postoperative PSA information were excluded from these analyses.

Postoperative PSA sensitivity analyses

Continuous postoperative PSA was evaluated using two time-updated measures. At the beginning of each interval, interval-maximum PSA was defined as the maximum PSA measured since prostatectomy if no previous MRI examination had occurred or since the preceding MRI examination otherwise. Latest-available PSA was defined as the most recent PSA measurement available at the beginning of the interval. PSA measurements performed on the same date as an MRI examination were not used for that examination because their temporal order could not be determined. Both models were fitted to the common set of intervals for which the two PSA measures were available.

Because PSA testing frequency differed across patients and potentially across hospitals, postoperative PSA was evaluated only in sensitivity analyses rather than included in the primary model. Postoperative PSA effect estimates were reported with additional decimal places to avoid masking small associations through rounding.

Temporal and proportional-hazards analyses

Annual crude MRI rates were calculated by dividing the number of MRI examinations in each postoperative year by the corresponding person-time and were expressed per 100 person-years. Exact Poisson 95% confidence intervals were calculated. Postoperative year 1 represented the period from prostatectomy to less than 1 year after surgery.

In the complementary Poisson model, postoperative time was modeled using a natural cubic spline with four degrees of freedom. Graphical analyses were restricted to postoperative years with at least 100 person-years of observation and at least 100 patients under follow-up.

Assessment of the proportional-hazards assumption

The proportional-hazards assumption of the primary counting-process Cox model was evaluated using scaled Schoenfeld residuals. Because the assumption was violated for hospital, an extended Cox model included interactions between hospital and postoperative time. The interaction terms were evaluated using a joint Wald test, and hospital-specific hazard ratios relative to Hospital 1 were estimated at postoperative years 1, 3, and 5.

Hospital-specific utilization and robustness analyses

Hospital-specific utilization was summarized using the numbers of patients and MRI examinations, total person-years, crude MRI rates per 100 person-years, and the proportions of patients undergoing zero, one, or two or more MRI examinations. Adjusted hospital-specific MRI rates were estimated from the Poisson model using standardization to the observed study population. Robustness was further assessed by sequentially excluding each hospital. Hospital 1 remained the reference institution when Hospitals 2–7 were excluded,

whereas Hospital 6 was used as the reference when Hospital 1 was excluded because it had the largest cohort among the remaining institutions.

Exploratory overall-survival analysis

Overall survival was measured from prostatectomy to death from any cause or the end of follow-up. Cumulative MRI use was included as a time-updated covariate in a separate counting-process Cox model. This analysis was considered exploratory and was not interpreted as estimating the causal effect of MRI use on survival.

Supplementary Table S1. Patient inclusion and reasons for exclusion according to hospital

Hospital	Initially eligible	Selection status		Reasons for exclusion, n			
		Included, n (%)	Excluded, n (%)	No preoperative PSA	No postoperative PSA	Interval between preoperative PSA and prostatectomy >1 year	No follow-up after prostatectomy
Hospital 1	2,388	2,093 (87.6)	295 (12.4)	258	33	4	0
Hospital 2	262	185 (70.6)	77 (29.4)	23	54	0	0
Hospital 3	284	229 (80.6)	55 (19.4)	51	3	1	0
Hospital 4	217	207 (95.4)	10 (4.6)	7	3	0	0
Hospital 5	429	324 (75.5)	105 (24.5)	74	27	3	1
Hospital 6	783	674 (86.1)	109 (13.9)	97	10	2	0
Hospital 7	44	42 (95.5)	2 (4.5)	1	1	0	0
**Total**	**4,407**	**3,754 (85.2)**	**653 (14.8)**	**511**	**131**	**10**	**1**

Percentages were calculated using the number of initially eligible patients at each hospital as the denominator.

PSA, prostate-specific antigen.

Supplementary Table S2. Comparison of available characteristics between included and excluded patients

**Characteristic**	**Included (n = 3,754)**	**Excluded (n = 653)**	***P* value**
Age at prostatectomy, years	70 (65–75)	70 (65–74)	0.328
Preoperative PSA, ng/mL	7.8 (5.3–13.7)	8.2 (5.1–13.0)	0.816
Interval from preoperative PSA measurement to prostatectomy, days	29 (2–62)	22 (1–64)	0.140
Grade group			0.969
1	462 (12.3)	84 (12.9)	
2	1,485 (39.6)	253 (38.7)	
3	1,082 (28.8)	194 (29.7)	
4	313 (8.3)	54 (8.3)	
5	412 (11.0)	68 (10.4)	
Pathological T stage			0.861
T1	23 (0.6)	3 (0.5)	
T2	2,273 (60.5)	407 (62.3)	
T3a	902 (24.0)	145 (22.2)	
T3b	540 (14.4)	95 (14.5)	
T4	16 (0.4)	3 (0.5)	

Data are medians (interquartile ranges) or numbers (percentages). Among excluded patients, preoperative PSA and the interval from preoperative PSA measurement to prostatectomy were unavailable in 511 patients; there were no missing observations for the other variables. Percentages for categorical variables were calculated using the total number of patients in each group as the denominator.

PSA, prostate-specific antigen.

Supplementary Table S3. Hospital-specific postoperative MRI utilization

Hospital	Patients	MRI examinations	Person-years	Crude rate per 100 person-years	Standardized rate per 100 person-years (95% CI)	No MRI, n (%)	One MRI, n (%)	Two or more MRIs, n (%)
Hospital 1	2,093	246	6707.1	3.67	3.69 (3.26–4.24)	1,878 (89.7)	190 (9.1)	25 (1.2)
Hospital 2	185	450	513.8	87.58	84.30 (77.85–90.98)	26 (14.1)	51 (27.6)	108 (58.4)
Hospital 3	229	141	796.0	17.71	17.07 (15.15–19.21)	99 (43.2)	119 (52.0)	11 (4.8)
Hospital 4	207	326	474.5	68.70	65.24 (57.47–74.44)	89 (43.0)	48 (23.2)	70 (33.8)
Hospital 5	324	442	828.7	53.34	52.38 (47.78–57.54)	135 (41.7)	68 (21.0)	121 (37.3)
Hospital 6	674	397	1942.6	20.44	20.83 (18.35–23.69)	381 (56.5)	237 (35.2)	56 (8.3)
Hospital 7	42	25	173.3	14.42	15.10 (11.00–20.64)	21 (50.0)	17 (40.5)	4 (9.5)

Rates are expressed per 100 person-years. Standardized rates were estimated from the adjusted Poisson model.

Supplementary Table S4. Assessment of the proportional-hazards assumption for the primary Cox model

Variable	Chi-square	Degrees of freedom	P value
Hospital	72.46	6	< 0.001
Preoperative PSA, per 5 ng/mL	0.38	1	0.538
Time-updated biochemical recurrence	0.13	1	0.714
Grade group	1.37	1	0.242
Pathological T stage	0.01	1	0.926
Age	0.04	1	0.84
Global	77.89	11	< 0.001

Scaled Schoenfeld residuals were calculated from the primary counting-process Cox model without continuous postoperative PSA.

Supplementary Table S5. Time-specific hospital hazard ratios from the extended Cox model

Hospital	Postoperative year 1 HR (95% CI)	Postoperative year 3 HR (95% CI)	Postoperative year 5 HR (95% CI)	*P* value for hospital-by-time interaction
Hospital 2	24.76 (20.93–29.29)	19.61 (14.97–25.68)	17.11 (11.20–26.14)	0.122
Hospital 3	3.13 (2.44–4.02)	6.32 (4.89–8.17)	9.53 (6.39–14.21)	< 0.001
Hospital 4	14.15 (11.56–17.32)	24.20 (18.20–32.17)	33.12 (21.65–50.68)	< 0.001
Hospital 5	11.37 (9.59–13.47)	19.79 (15.41–25.40)	27.37 (18.64–40.18)	< 0.001
Hospital 6	5.12 (4.25–6.17)	6.44 (4.89–8.49)	7.36 (4.88–11.12)	0.100
Hospital 7	2.43 (1.35–4.37)	5.67 (3.77–8.54)	9.31 (4.81–18.04)	0.009

Hospital 1 was the reference institution. Estimates were obtained from the extended Cox model containing interactions between hospital and log(1 + postoperative time). The interaction terms were jointly significant (Wald $\chi^2 = 87.23$; df = 6; $P< 0.001$). The individual P values represent the hospital-by-time interaction for each hospital relative to Hospital 1.

HR, hazard ratio; CI, confidence interval.

Supplementary Table S6 Annual postoperative MRI utilization rates

Postoperative year	Patients under follow-up	MRI examinations	Person-years	MRI rate per 100 person-years (95% CI)
1	3,754	557	3460.5	16.10 (14.79–17.49)
2	3,022	629	2590.3	24.28 (22.42–26.26)
3	2,248	370	1917.0	19.30 (17.38–21.37)
4	1,627	238	1397.8	17.03 (14.93–19.33)
5	1,186	175	968.1	18.08 (15.50–20.96)
6	707	34	572.5	5.94 (4.11–8.30)
7	431	14	334.0	4.19 (2.29–7.03)
8	233	5	157.3	3.18 (1.03–7.42)

Postoperative year 1 denotes 0 to <1 year after prostatectomy. Only years with at least 100 person-years and 100 patients under follow-up are shown.

Supplementary Table S7. Leave-one-hospital-out sensitivity analysis

Excluded hospital	Hospital 2	Hospital 3	Hospital 4	Hospital 5	Hospital 6	Hospital 7
**Analyses retaining Hospital 1 as the reference institution**						
Hospital 2	—	4.629 (3.859–5.553)	18.143 (14.912–22.074)	14.679 (12.464–17.287)	5.664 (4.704–6.820)	4.077 (2.895–5.740)
Hospital 3	23.075 (19.716–27.006)	—	17.956 (14.801–21.784)	14.475 (12.302–17.032)	5.623 (4.674–6.766)	4.099 (2.915–5.766)
Hospital 4	22.886 (19.553–26.789)	4.615 (3.853–5.528)	—	14.265 (12.117–16.793)	5.613 (4.664–6.755)	4.098 (2.918–5.755)
Hospital 5	22.997 (19.645–26.920)	4.639 (3.873–5.556)	17.658 (14.508–21.492)	—	5.665 (4.705–6.820)	4.089 (2.914–5.737)
Hospital 6	23.111 (19.728–27.073)	4.639 (3.870–5.560)	17.838 (14.670–21.690)	14.512 (12.332–17.077)	—	4.079 (2.898–5.741)
Hospital 7	23.117 (19.756–27.049)	4.636 (3.870–5.554)	17.998 (14.845–21.821)	14.462 (12.296–17.009)	5.648 (4.697–6.792)	—
**Analysis excluding Hospital 1 with Hospital 6 as the reference institution**						
Hospital 1	4.080 (3.526–4.720)	0.810 (0.681–0.962)	3.288 (2.767–3.907)	2.521 (2.175–2.923)	Reference	0.703 (0.503–0.983)

Values are adjusted hazard ratios (95% confidence intervals) from counting-process Cox models with patient-clustered robust variance estimates. In the upper section, each nonreference hospital was excluded in turn while Hospital 1 remained the reference institution. In the lower section, Hospital 1 was excluded and Hospital 6 was used as the reference because it had the largest cohort among the remaining institutions. After Hospital 1 was excluded, the overall hospital effect remained significant (robust Wald $\chi^2 = 759.03$; df = 5; $P < 0.001$). All models included preoperative PSA, time-updated biochemical recurrence, grade group, pathological T stage, and age. CI, confidence interval; HR, hazard ratio; PSA, prostate-specific antigen.

Supplementary Table S8. Exploratory overall-survival model

Variable	HR (95% CI)	P value
Cumulative MRI examinations	0.782 (0.564–1.086)	0.142
Hospital 1	Reference	—
Hospital 2	1.122 (0.300–4.205)	0.864
Hospital 3	0.231 (0.055–0.969)	0.045
Hospital 4	0.710 (0.239–2.113)	0.538
Hospital 5	0.803 (0.340–1.897)	0.618
Hospital 6	0.520 (0.261–1.034)	0.062
Hospital 7	0.459 (0.058–3.640)	0.461
Preoperative PSA, per 5 ng/mL	1.011 (1.001–1.022)	0.033
Biochemical recurrence	4.562 (2.672–7.789)	< 0.001
Grade group ≥2 vs 1	3.017 (0.934–9.749)	0.065
Pathological T stage ≥T3 vs T1–T2	1.077 (0.720–1.613)	0.717
Age, per year	1.082 (1.042–1.124)	< 0.001

This analysis was exploratory and was not designed to estimate a causal effect of MRI utilization on survival. The overall joint test of the hospital coefficients was not statistically significant (robust Wald $\chi^2 = 7.86$; df = 6; $P = 0.249$). CI, confidence interval; HR, hazard ratio; PSA, prostate-specific antigen.

Supplementary Figure S1. Time-varying hospital hazard ratios for repeated postoperative MRI use.

Hospital-specific hazard ratios relative to Hospital 1 over postoperative time were estimated from the extended Cox model containing hospital-by-time interactions. Shaded areas indicate 95% confidence intervals, and the horizontal dashed line indicates a hazard ratio of 1.

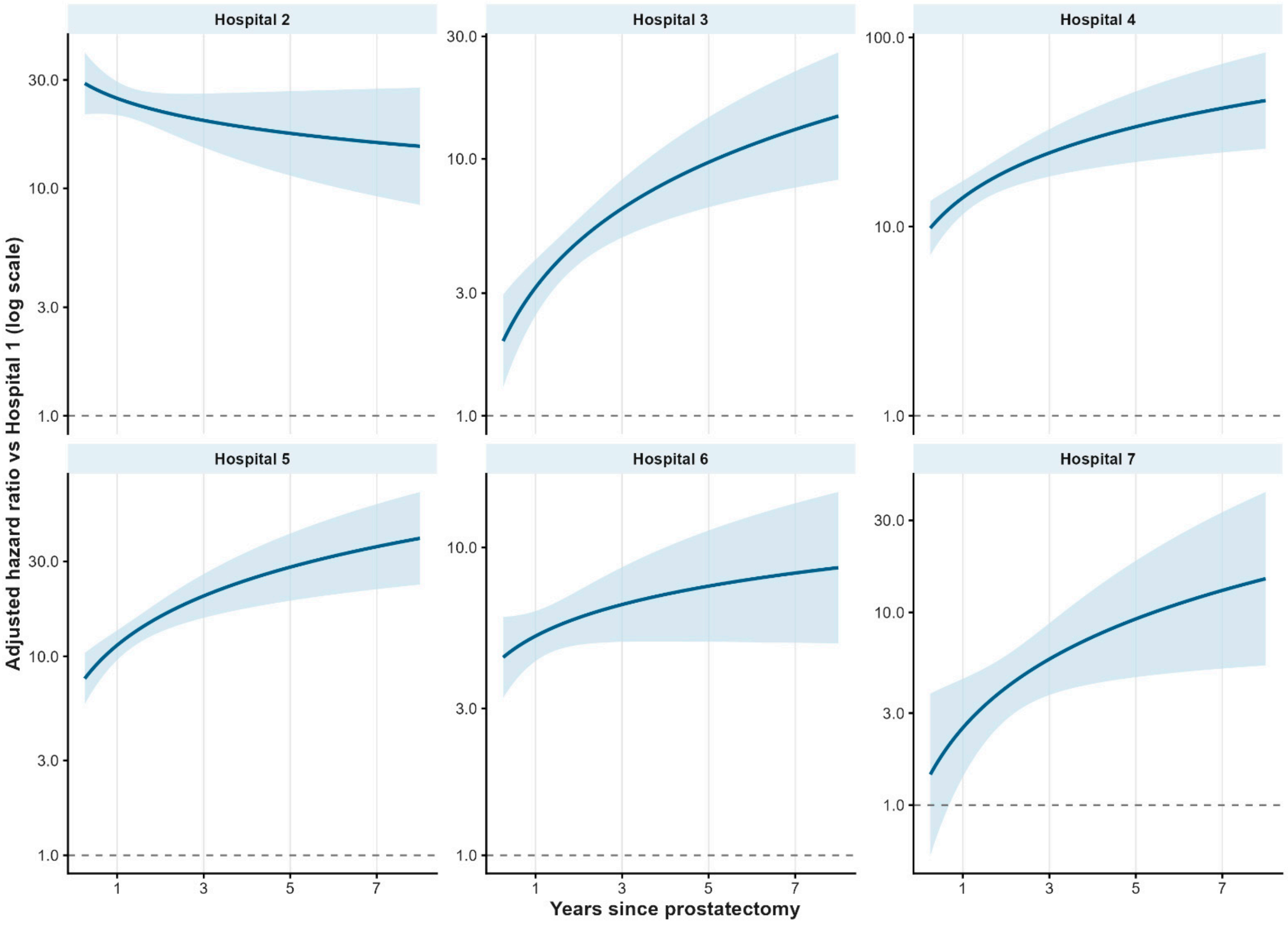

Supplement: Supplementary file 1 [file healthcare-14-03131-s001.zip › healthcare-4461541-supplementary.pdf]
